# Metastatic heterogeneity of the consensus molecular subtypes of colorectal cancer

**DOI:** 10.1038/s41525-021-00223-7

**Published:** 2021-07-14

**Authors:** Peter W. Eide, Seyed H. Moosavi, Ina A. Eilertsen, Tuva H. Brunsell, Jonas Langerud, Kaja C. G. Berg, Bård I. Røsok, Bjørn A. Bjørnbeth, Arild Nesbakken, Ragnhild A. Lothe, Anita Sveen

**Affiliations:** 1 Department of Molecular Oncology, Institute for Cancer Research, Oslo University Hospital, Oslo, Norway; 2 K.G. Jebsen Colorectal Cancer Research Centre, Division for Cancer Medicine, Oslo University Hospital, Oslo, Norway; 3 Institute for Clinical Medicine, University of Oslo, Oslo, Norway; 4 Department of Gastrointestinal Surgery, Oslo University Hospital, Oslo, Norway

**Keywords:** Colorectal cancer, Transcriptomics, Tumour heterogeneity, Bioinformatics, Tumour biomarkers

## Abstract

Gene expression-based subtypes of colorectal cancer have clinical relevance, but the representativeness of primary tumors and the consensus molecular subtypes (CMS) for metastatic cancers is not well known. We investigated the metastatic heterogeneity of CMS. The best approach to subtype translation was delineated by comparisons of transcriptomic profiles from 317 primary tumors and 295 liver metastases, including multi-metastatic samples from 45 patients and 14 primary-metastasis sets. Associations were validated in an external data set (*n* = 618). Projection of metastases onto principal components of primary tumors showed that metastases were depleted of CMS1-immune/CMS3-metabolic signals, enriched for CMS4-mesenchymal/stromal signals, and heavily influenced by the microenvironment. The tailored CMS classifier (available in an updated version of the R package CMScaller) therefore implemented an approach to regress out the liver tissue background. The majority of classified metastases were either CMS2 or CMS4. Nonetheless, subtype switching and inter-metastatic CMS heterogeneity were frequent and increased with sampling intensity. Poor-prognostic value of CMS1/3 metastases was consistent in the context of intra-patient tumor heterogeneity.

## Introduction

Gene expression-based subtypes of colorectal cancer (CRC) have important clinical associations, potentially also with response to anticancer agents in the metastatic setting^1^. However, the most widely adopted transcriptomic classification framework (the consensus molecular subtypes, CMS)^2^ was initially developed for primary tumors, and there are strong indications of contextual variation. Metastatic CRCs likely represent a skewed distribution of the CMS groups. This expectation is partly based on prognostic associations, in particular, the poor prognosis associated with primary CMS4-mesenchymal/stromal tumors; the different prevalence of CMS-associated genetic markers between primary and metastatic CRCs, including microsatellite instability (MSI) and *BRAF*^V600E^/*KRAS* mutations^3,4^; as well as the effect of systemic therapy^5^. Despite strong signaling enrichments with targets of standard-of-care treatments among CMS groups, two recent retrospective analyses of randomized clinical trials evaluating first-line combination chemotherapies with either anti-EGFR or anti-angiogenic agents against metastatic CRCs, showed discordant results for the predictive value of CMS^6,7^. Accordingly, CMS classification is in its current form not ready for clinical translation, and even the representativeness of the CMS framework for heterogeneous metastatic cancers is unknown^8^. There is a need for a thorough inquiry into the extent to which subtypes are maintained over time, across tumor sites, and between metastatic lesions from the same patient (longitudinal and spatial intra-patient heterogeneity).

A complicating factor of such interrogations is the fact that gene expression profiles of bulk tumor tissue samples represent the sum of signals from the cancerous cells and tumor microenvironment^9,10^. These signals are biologically interlinked, but variation in their relative abundances is seen among samples from different tumor regions^11,12^. Furthermore, variation in the nature of microenvironment signals is expected between samples from the primary and metastatic sites. We and others have previously provided a solution to the analogous problem of classifying pre-clinical models, which either lack a tumor microenvironment entirely (cell lines and organoid cultures), or present a completely different background (murine xenografts)^13,14^. This indicates the feasibility of robust CMS classification in the context of a changing tumor microenvironment.

We aimed to investigate the biological and prognostic associations of CMS in the context of metastatic tumor heterogeneity. To delineate the optimal approach to the translation of the classification to metastases, we initially explored the potential skewedness of transcriptomic profiles between primary and metastatic tumors.

## Results

### CMS framework captured by principal components

**Fig. 1:**
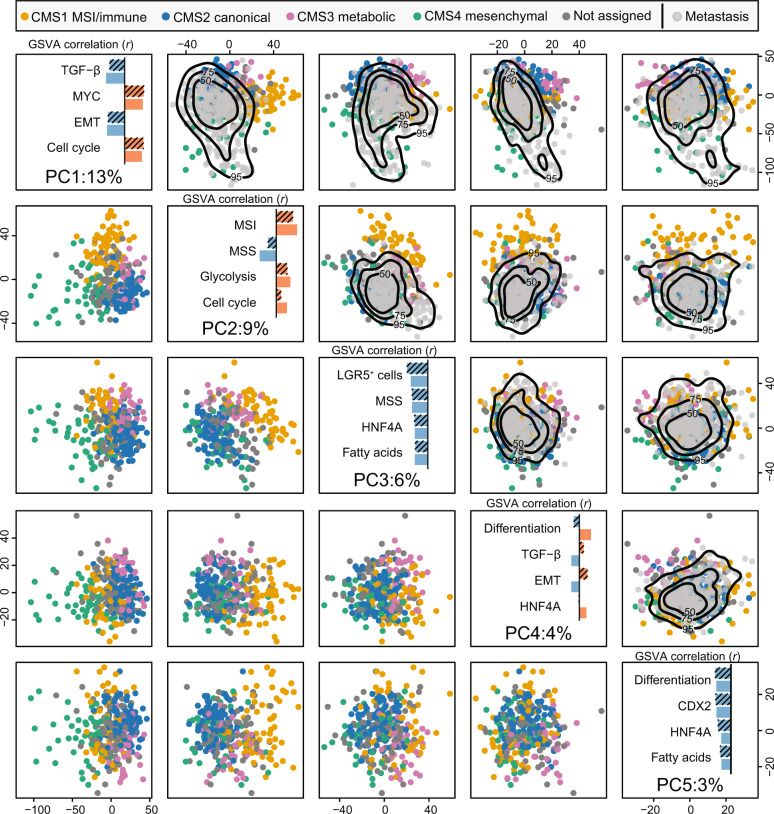
Liver metastases show reduced variation along the principal components that separate CMS1 and CMS3 primary tumors from CMS2 and CMS4. The lower triangle of the scatterplot matrix depicts the first five principal components (PCs) for primary CRCs, with samples colored according to CMS (dark gray = not confidently classified). The diagonal windows show the percentage of total variance explained by each PC (bottom) and the Pearson correlations (*r*) between the indicated PC and single-sample gene set scores (GSVA) for the top-4 correlated gene sets among primary tumors (hatched bars; blue and red represent negative and positive correlations, respectively). Correlations among liver metastases are shown for comparison (non-hatched bars). The upper triangle represents the transpose of the lower triangle with the metastatic samples (light gray) superimposed by projections onto the same PCs. The black contour lines represent the 50%, 75%, and 95% two-dimensional density estimates. The presented data are from the in-house series, and results from the same analysis of the external validation data set are included in Supplementary Fig. 2.

To systematically compare CRC gene expression profiles in the primary and metastatic settings in relation to CMS, we performed exploratory analyses of an in-house data set of 317 primary tumor samples (315 patients) and 295 liver metastasis samples (176 patients; Table 1). The first five principal components (PC1-PC5) defined by principal component analysis (PCA) of the primary tumors had significantly different medians across the CMS groups (*p* = 2 × 10^−4^, Kruskal–Wallis test; Supplementary Fig. 1a), illustrating the association between CMS and global differences in gene expression of primary tumors (Fig. 1, left lower half of the matrix). Single-sample gene set variation analysis (GSVA^15^) of 14 pre-selected and CRC-informative gene sets showed that PC1 was most strongly correlated with the TGF-β and EMT signatures, and inversely with MYC and cell cycle signatures (absolute Pearson correlation coefficient |*r*| > 0.64). PC1-scores also separated microenvironment-dominated CMS1/CMS4 primary tumors from more epithelial-like CMS2/CMS3 tumors. Eighty-eight (77%) of 114 tumors with PC1 > 0 were either CMS1 or CMS4, while this was the case for only 16 (12%) of the 138 tumors with PC1 < 0 (odds ratio [OR] = 25 [95% confidence interval, CI: 12–54], *p* < 2 × 10^−16^). PC2 was most strongly correlated with the MSI and microsatellite stable (MSS) transcriptomic signatures (|*r*| > 0.61), and separated the predominantly MSS subtypes CMS2/CMS4 from MSI-enriched CMS1/CMS3, consistent with strong enrichment for primary tumors with positive MSI status among samples with a high PC2-score (58 MSI of 128 with PC2 > 0; 1 MSI of 166 with PC2 < 0, OR > 134, *p* < 2 × 10^−16^). PC3 was correlated with the intestinal LGR5^+^ stem-cell signature (|*r*| = 0.63), and separated CMS2/CMS4 from CMS1/CMS3 (at PC3 > 0 threshold: OR = 7.4 [95% CI 4–14], *p* = 1 × 10^−12^), similarly to PC2. PC4 and PC5 were both associated with the gastro-intestinal differentiation signature (|*r*| > 0.42), and distinguished CMS3 from the remaining subtypes. Largely concordant results were found among primary CRCs from an independent data set (*n* = 545^5^; Supplementary Fig. 2).

**Table 1:** Clinicopathological characteristics.

	Primary CRC (*n* = 315 patients^a^)		Metastatic CRC (*n* = 176 patients^a^)	
	No. of patients	%	No. of patients	%
Age at surgery				
Below 65 years	94	30	81	46
65–75 years	105	33	76	43
Above 75 years	116	37	19	11
Gender				
Male	169	54	110	62
Female	146	46	66	38
Primary tumor location				
Left/rectum	181	57	133	76
Right	126	40	35	20
Synchronous	7	2	1	1
Unknown	1	0.3	7	4
Cancer stage at first diagnosis				
I	65	21	2	1
II	117	37	15	9
III	90	29	112	64
IV	41	13	31	18
Unknown	2	1	16	9
Diagnosis of liver metastasis				
Synchronous (within 6 months of initial diagnosis)	–	–	138	78
Metachronous	–	–	38	22
Systemic oncological treatment prior to sampling of metastases				
Neoadjuvant chemotherapy for this metastatic situation	–	–	134	76
Previous chemotherapy only	–	–	27	15
No previous systemic treatment (naive)	–	–	15	9

^a^Fourteen patients with matched primary and metastatic tumor samples.

### Transcriptomic selection of resected liver metastases compared to primary CRCs

To indicate whether gene expression variation captured by CMS in the primary setting would be sensitive to expression signals enriched in liver metastases (due to a different tumor microenvironment), we calculated a sample-wise “liver background” score based on genes with hepatocyte-enriched expression (Human Protein Atlas^16^). As expected, this score was not correlated with the first five PCs among primary CRCs, and explained only a negligible amount of cross-sample variance (*r*^2^ < 0.11; Supplementary Fig. 1b). The liver metastases were therefore added onto the same five projections (PC1-PC5) defined by the primary tumors. This illustrated that liver metastases largely occupied the same high-variance transcriptomic subspace as primary CRCs (Fig. 1, right upper half of the matrix). The biological validity of the projection was confirmed by similar Pearson correlations between PC scores and gene signatures in the primary and metastatic tumors (Fig. 1, diagonal windows). However, striking differences in sample distributions were also seen, and PC1 among metastases was significantly shifted towards the space populated by primary CMS4 tumors (*p* < 2 × 10^−16^, Wilcoxon test; Supplementary Fig. 1), indicating a strong enrichment with CMS4 in the metastatic setting. Furthermore, the interquartile range of PC2 scores was much smaller among metastases than primary tumors (21 versus 30), indicating a relative depletion of samples with MSI-like gene expression characteristics, predominantly CMS1/CMS3 tumors, in the metastatic setting. There were no metastatic samples with very high PC4 scores, again indicating depletion of CMS3. These results were corroborated by DNA-level analyses, and the prevalence of MSI was 20% among primary tumors and only 0.6% among metastases (calculated patient-wise: 1 of 176 patients). Importantly, this skewed transcriptomic distribution of metastases relative to primary tumors was also found in the external validation data set of 545 primary tumors and 73 metastases (Supplementary Fig. 2).

Notably, the first two components from in-house primary tumors were highly similar to previously published PCA-derived subtype scores of primary CRCs^17^ (|*r*| > 0.78; Supplementary Fig. 3), supporting that PCA separates the primary, and by extension, the metastatic tumors according to the CMS framework.

### Translation of CMS classification to the metastatic setting

**Fig. 2:**
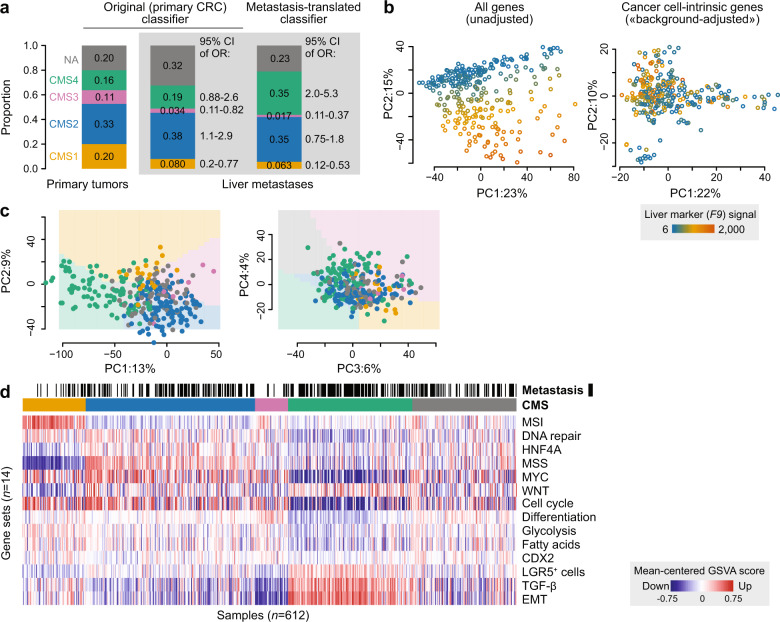
Translation of CMS classification to liver metastases. **a** Barplots illustrate differences in CMS distributions between primary tumors and liver metastases using the original primary CRC classifier and the new metastases-translated classifier. Odds ratios (ORs) indicate changes in proportions of each CMS compared to the primary setting (only counting confidently classified samples). **b** Principal component analyses demonstrate that the transcriptomic “liver background” (here illustrated by expression of the hepatocyte-enriched F9 gene marker) represents a large source of variation in raw expression data from bulk tissue samples of liver metastases (left), but not when only considering the 1104 cancer cell-intrinsic candidate markers after “liver background-adjustment” (right). **c** Principal component analyses showing CMS-classified liver metastases (colored dots, *n* = 295) projected onto the first four principal components from primary tumors. Colored backgrounds represent the dominant subtype in the primary tumors. **d** Gene set analysis with sample type (black: metastases; white: primary tumors) and assigned CMS (as in **a**) indicated on top. Samples are ordered according to CMS and p-values from the tailored CMS classifier. Gene sets (in rows) are ordered according to CMS hallmarks (CMS1 characteristics first, followed by CMS2 and so on).

Application of the original random forest CMSclassifier^2^ to the liver metastases suggested that the transcriptomic selection was partly reflected also in CMS classification. Compared to the primary CRCs, there was a significant depletion of both CMS1 and CMS3 tumors (95% CI of OR 0.20–0.77 and 0.11–0.82, respectively), and an enrichment with CMS2 (95% CI of OR 1.1–2.9) among the confidently classified metastases (*n* = 176 patient-wise unique samples; Fig. 2a). However, there was a much larger proportion of unclassified samples among the metastases (32% versus 20%), and there was no enrichment with CMS4 tumors. This suboptimal performance of the original classifier was likely attributed to violation of the assumption that the query data should have a similar distribution in gene expression values as the training data (represented by liver metastases and primary tumors, respectively, in Fig. 1). This violation might be caused by sample selection inherent to the metastatic process, variation caused by different expression signals from the tissue backgrounds (Fig. 2b), and exposure to chemotherapy (Supplementary Fig. 4). We, therefore, developed a tailored classifier for liver metastases, adopting our approach previously used to classify pre-clinical models^13^. We first performed a heuristic feature selection among protein-coding genes (to maximize cross-platform portability) in CRC cell lines and patient-derived organoids (to minimize sensitivity to microenvironment signals), leaving 1104 genes as features for classification (Methods). This template gene set was used to train a “nearest shrunken centroids” classifier^18^ on gene expression data from primary CRCs with a known CMS label (*n* = 252 confidently classified tumors, *t* = 1.5, cross-validation accuracy = 0.93, Supplementary Fig. 5). The resulting classifier was then applied to liver metastases from the in-house series (*n* = 295 lesions from 176 patients), after adjusting for gene expression signals from the “liver background” in each sample by regression analyses (Fig. 2b and “Methods” section). This provided classifications in accordance with expectations from exploratory analyses (Fig. 2a). Specifically, we recorded strong depletion of CMS1 and CMS3 tumors relative to the primary setting (95% CI of OR 0.12–0.53 and 0.011–0.37, respectively), and an enrichment with CMS4 (95% CI of OR 2.0–5.3), while the proportion of CMS2 tumors was similar to the primary setting (95% CI of OR 0.75–1.8).

The overall concordance of confidently classified liver metastases between the original and tailored classifier was 0.93 (Supplementary Fig. 5d), but the tailored classifier resulted in fewer unclassified samples (23% versus 32%), and the expected enrichment with CMS4 was only significant with the new classifier translated to the metastatic setting. Notably, 205/230 (89%) of the confidently classified liver metastases were either CMS2 or CMS4.

### CMS associations recapitulated among liver metastases

Projection of CMS-classified liver metastases onto PC1–PC4 from primary tumors showed concordance between assigned subtypes and global expression profiles (Fig. 2c). To further compare CMS characteristics between the primary and metastatic settings (classification with the original and tailored classifier, respectively), we used single-sample gene set analyses as above (Fig. 2d and Supplementary Fig. 6). Notably, the most pronounced discordance was independent of CMS, and involved generally lower scores among metastases for the cell cycle, DNA repair, and MYC signatures (*p* < 1 × 10^−8^, Wilcoxon rank-sum test). CMS associations were strongly correspondent between primary and metastatic tumors. For example, EMT and TGF-β signals were higher in CMS4 in both settings, and the gene expression-based MSI score was highest in CMS1. The single patient with MSI-high liver metastases was clearly CMS1. However, no metastases were among the most prototypical CMS1 or CMS3 tumors, and the least convincing pattern was the failure to capture enrichment with metabolic signals in CMS3. The latter might be explained by the small number of CMS3 metastases, but weak metabolic signaling in CMS3 was common to the primary and metastatic settings.

Few (3%) of the metastases had a *BRAF*^V600E^ mutation, but the highest proportion was found in CMS1 (7%; Supplementary Fig. 7a). The distribution of *TP53* mutations was significantly skewed among CMS groups, with the highest proportion in CMS2 (86%, *p* = 0.0005, Fisher’s test; Supplementary Fig. 7b), similarly to the original publication of primary CRCs^2^. Co-occurrence of *BRAF*/*KRAS*/*NRAS* and *TP53* mutations was significantly enriched in CMS1 and CMS3 (50% and 56%) compared to CMS2 and CMS4 (27% and 28%; *p* = 0.006, Fisher’s test; Supplementary Fig. 7c). Right-sided primary tumor location was most frequent in CMS1, although “tumor sidedness” (right versus left + rectum) was not significantly different among the CMS groups, potentially related to the low prevalence of CMS1 among liver metastases (Supplementary Fig. 7d).

The potential impact of chemotherapy exposure prior to tumor sampling (Supplementary Fig. 4) was not adjusted for in the tailored CMS classifier, based on the rationale that previous treatment exposure will be relevant in most analysis settings. There was a strong enrichment with CMS4, and a corresponding depletion of CMS2, in samples exposed to neoadjuvant chemotherapy compared to the few samples with no or only previous treatment exposure (*p* = 0.0005, Fisher’s test; Supplementary Fig. 8a). Gene set analyses further showed that samples exposed to neoadjuvant chemotherapy had lower cell cycle, MSS, and MYC signature scores, both in the analysis of all and of only CMS4 samples (Supplementary Fig. 8b, c). EMT and TGF-β signatures were significantly higher in the neoadjuvant treatment-group, consistent with CMS4-enrichment. However, the EMT signature was also significantly higher when analyzing CMS4 samples only, suggesting that CMS4 characteristics were largely similar with and without neoadjuvant treatment exposure. Furthermore, important CMS associations were found in separate analyses of the subset of patients who did not receive neoadjuvant chemotherapy (Supplementary Fig. 8d). This included high MSI scores in CMS1, high DNA repair and HNF4A signaling in CMS2 and CMS3, high glycolysis scores in CMS3, as well as strong EMT and TGF-β signals in CMS4.

### Pronounced intra-patient CMS heterogeneity in the metastatic setting

**Fig. 3:**
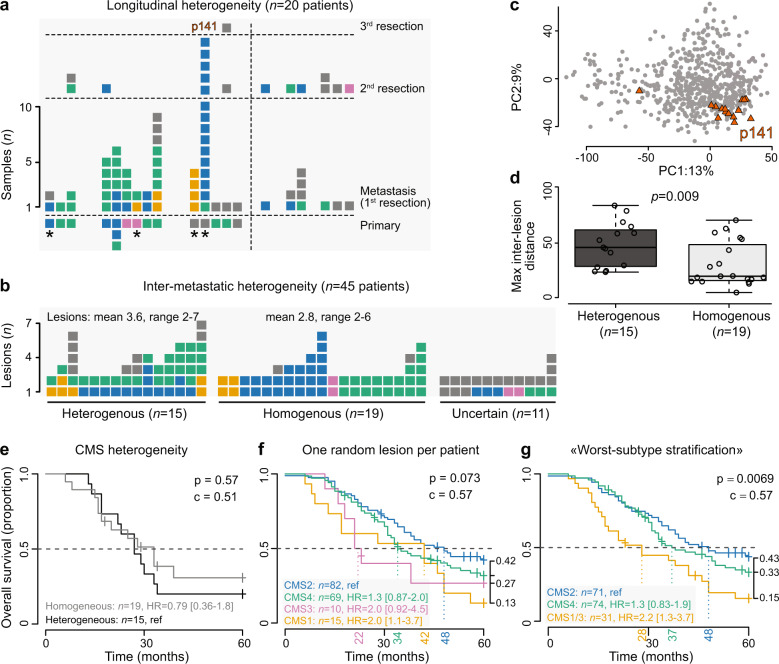
Pronounced CMS heterogeneity in metastatic CRC. Stacked barplots show intra-patient heterogeneity (each bar represents one patient and each square represents one tumor sample) of CMS assignments for patients with **a** matched primary tumors (classified with the original classifier)/longitudinal samples and liver metastases (classified with the new, tailored classifier; asterisk indicates patients with no neoadjuvant treatment), and **b** multiple resected metastatic lesions. **c** PCA plot illustrating transcriptomic heterogeneity for one patient with heterogeneous CMS classifications (p141), where most lesions are relatively similar and classified in the same CMS group, but with a single strong outlier sample with a different classification. **d** Boxplots (center line, median; box limits, upper and lower quartiles; whiskers, 1.5 × interquartile range) where patients with heterogeneous and homogeneous CMS classifications are compared with respect to maximum inter-lesion distance, calculated as Euclidean distances in the three-dimensional PC1–PC3 space. Kaplan–Meier survival estimates for patients with resected liver metastases, stratified by **e** inter-metastatic CMS heterogeneity, **f** CMS classification based on one randomly selected lesion per patient, and **g** “worst-subtype stratification” according to the following rule: if any lesion CMS1/CMS3, then patient CMS1/CMS3; if no CMS1/CMS3 and any lesion CMS4, then patient CMS4; if all CMS2, then patient CMS2. *p*-values were calculated by Wald tests, c is the concordance index, and HR is the hazard ratio. For survival analyses, each sample is assigned to the nearest (not necessarily confident) CMS.

Comparisons of matched primary and metastatic tumors from 14 patients (76 samples from 59 lesions) showed that only three patients were assigned a single CMS (Fig. 3a; uncertain concordance status in 5 patients due to unclassified samples). There was no apparent difference in the pattern or rate of subtype switching between patients who received neoadjuvant chemotherapy for their liver metastases (subtype switching in 5 of 7) and patients who did not (1 of 2). Subtype switching was also seen between the first and second liver resections in 4 of the 6 evaluable patients.

Multiple distinct metastatic lesions from the same liver resection were compared for 45 patients (2–7 lesions per patient). Among patients with more than one confidently classified lesion (*n* = 34), intra-patient inter-metastatic CMS heterogeneity was found in 44% (Fig. 3b). Heterogeneity was associated with a higher number of lesions analyzed (*p* = 0.004, Wilcoxon rank-sum test). A particularly striking example was patient 141 (Fig. 3c) with 15 samples from two separate metastatic resections (5 and 3 lesions, respectively), for whom 14/15 samples were classified as CMS2, while the remaining lesion was CMS4 (the primary tumor also resembled CMS4, but was not confidently classified). Some of this intra-patient heterogeneity may be attributed to ambiguity near the class boundaries, but analyses of general patient-wise transcriptomic heterogeneity (Methods) supported the heterogeneous classifications. The largest maximum Euclidean distance between any two samples was higher for patients with heterogeneous classifications (Fig. 3d). The degree of intra-patient inter-metastatic transcriptomic heterogeneity was not different between patients who did or did not receive neoadjuvant chemotherapy (Supplementary Fig. 9).

Heterogeneous intra-patient inter-metastatic CMS classification was not associated with patient survival (Fig. 3e). However, the translated CMS framework did show prognostic potential, and patient stratification based on a randomly selected metastasis per patient showed shorter median overall survival for CMS1 and CMS3 compared to CMS2 and CMS4 (*p* = 0.07, Wald-test; Fig. 3f). Given the extent of intra-patient CMS heterogeneity, we subsequently performed “worst-subtype stratification” according to the following rule: if any lesion CMS1/CMS3, then patient CMS1/3 (combining the two poor-prognostic subtypes); if no CMS1/3 and any lesion CMS4, then patient CMS4; otherwise CMS2. With this stratification, the median overall survival was 28, 37, and 48 months, respectively, showing a significantly worse survival for patients with any tumor classified in the MSI-like CMS1/CMS3 groups (*p* = 0.007, Wald-test; Fig. 3g). This was independent of neoadjuvant chemotherapy in a stratified analysis (*p* = 0.004, Wald-test). For comparison with previously published studies, the analyses were repeated with classifications based on the original primary CRC classifier, showing similar results (Supplementary Fig. 10).

## Discussion

We have translated CMS classification to CRC liver metastases based on the delineation of transcriptomic skewedness between primary and metastatic tumors, and implementation of a computational approach to regress out signals from the tissue background in gene expression profiles of bulk tumor tissue samples. The tumor microenvironment has a confounding effect on classification concordance of patient-matched primary and metastatic samples^19^, and this study provides a dedicated adaptation of CMS classification to metastatic CRCs. The principal benefit with the tailored classifier was indeed a more robust classification of the microenvironment-rich CMS4-mesenchymal metastases. This supports the strategy of using CMS-associated expression signals intrinsic to cancer cells to classify tumors originating from different sites, and is consistent with our previous results showing the potential to classify also pure CRC cell cultures^13^. The tailored subtyping algorithm is available in an updated version of the R package CMScaller (v2.0.1), and includes build-in functionalities such as gene set analyses to evaluate the classification.

Known biological characteristics of the CMS framework were recapitulated in the metastatic setting. However, almost 90% of the liver metastases belonged to one of only two subtypes, either CMS2-canonical or CMS4-mesenchymal, suggesting that the classification framework has a weaker biological discriminatory power in metastatic compared to primary CRCs. This is in accordance with previous studies showing a strong relative depletion of CMS1-MSI/immune and CMS3-epithelial/metabolic subtypes among metastases, although based on analyses with the original CMS classifier^3,5^. The difference in subtype distribution between the two disease settings is likely attributed to a combination of biological and extrinsic factors, including variation in metastatic propensity and treatment exposure, respectively. Metastatic propensity is higher with CMS4-mesenchymal primary tumors and lower with MSI-related subtypes (CMS1 and, to a lesser extent, CMS3)^2^. In addition, some CMS-associated characteristics such as MSI status, *BRAF*^V600E^, and *KRAS* mutations, as well as primary tumor location, are associated with different patterns of metastatic spread. These characteristics, therefore, have a different distribution among metastatic sites^20,21^, as well as between resectable and non-resectable disease^22^. This has likely contributed to a particularly high CMS4 versus CMS1/CMS3 ratio in our study, which included resected liver metastases, representing a selected subset of metastatic CRCs. Furthermore, chemotherapy is frequently given prior to resection and sampling of metastatic tumors, and has been shown to associate with a shift towards a more mesenchymal phenotype^5,23^. Most of the samples in our study were exposed to systemic neoadjuvant treatment, and this was associated with a strong enrichment with CMS4, in agreement with published data^24^. Combination chemotherapy is part of the first-line treatment of most patients with metastatic CRC, and the choice not to adjust for systemic therapy was based on the rationale that prior treatment exposure will be relevant in most studies in which the tailored CMS classifier is applicable, including clinical studies of experimental therapies.

The strong interest in the investigation of CMS in metastatic CRC has to some extent been driven by its potential treatment prediction value, including the biological rationale for differential sensitivity to standard anticancer agents targeting EGFR or VEGF. A strong relative sensitivity to EGFR inhibition in CMS2-like subtypes has been supported in preclinical studies, both with in vitro and in vivo models^25,26^. However, the lack of reproducible results for associations with clinical treatment responses^6,7^ is likely partly attributed to tumor heterogeneity^8^. Intra-patient CMS heterogeneity was frequently observed in our study, and although the comparison of matched primary tumors and metastases was based on a small set of patients, the frequent subtype switching was in line with previous studies showing a discordance rate of ~40% in CRCs metastasizing to the liver^19,24^. Accordingly, the use of primary tumor gene expression profiles as a basis for CMS classification in the clinical trial cohorts likely impacted the analyses of associations to anti-EGFR versus anti-VEGF therapy response. Furthermore, phenotypic plasticity provides an alternative pathway to drug tolerance^27^, and we also observed evidence of subtype switching between repeated hepatic resections. An evolutionary shift from CMS2 to CMS4 characteristics after anti-EGFR therapy has been shown to associate with the development of secondary treatment resistance in metastatic CRC^28^. The collective evidence of CMS plasticity in relation to selection pressures, such as metastasis and exposure to chemotherapies or targeted agents, highlights a potentially decisive role of the timing and site of tumor sampling for accurate analysis of CMS in relation to clinical endpoints. Finally, we provide strong evidence of frequent subtype heterogeneity also among distinct liver lesions from individual patients. In fact, the results show that more heterogeneity should be expected with more intense sampling, supporting that there is an upper limit to the accuracy of CMS biomarker status attainable from a single sample. Classification heterogeneity was also shown to be associated with general transcriptomic heterogeneity, suggesting that this is an inherently difficult feature to overcome. Few if any transcriptomic features of CRC show an “on-off” pattern and it has been suggested that subtype distinctions may be of a continuous nature^17,29^. Notably, the CMS framework did capture the biology represented by such “continuous subtypes” in liver metastases. However, more refined gene expression profiling methods like single-cell RNA sequencing^30^ and spatial analyses^31^, or alternatively image-based methods^32–34^, might improve the foundation to resolve this challenge.

Despite challenges with tumor heterogeneity and dependence on the tumor microenvironment, the CMS framework has repeatedly been shown to have prognostic value also in the metastatic setting. Metastatic CMS1 and to a lesser extent CMS3 cancers are associated with a particularly poor patient survival^6,7,19^. This is partly explained by the aggressiveness of the MSI phenotype in the metastatic setting, but was also found in our study, where 99% of patients had MSS liver metastases. The majority of patients with resected liver metastases classify as either CMS2 or CMS4, and for these patients, the prognostic discriminatory power seems weaker, although it should be noted that the small and not statistically significant survival difference indicating a benefit with CMS2 tumors has been shown consistently across studies^6,7,19^. Importantly, the poor-prognostic value of CMS1/CMS3 was in our study maintained in the context of tumor heterogeneity, and patients with at least one CMS1 or CMS3 metastasis had the shortest median overall survival. The CMS framework identified 18% of the patients to belong to this poor-prognostic subgroup when multiple sampling was performed, but the patient number was limited and larger studies are needed to conclude on the requirements for sample numbers per patient, as well as the prognostic value relative to clinicopathological factors and other prognostic biomarkers.

In conclusion, we have developed a method to translate CMS classification to the metastatic setting. Nearly 90% of resected and frequently chemotherapy-exposed liver metastases belonged to either the CMS2 or CMS4 groups. Intra-patient CMS heterogeneity was pronounced and increased with sampling intensity, suggesting that the underlying heterogeneity might be even larger than estimated in this study. Metastatic heterogeneity, therefore, represents a challenge to the usefulness of the CMS framework, potentially associated with the continuous rather than discrete nature of subtype distinctions. However, clear biological distinctions of at least the most “subtype-typical” samples, as well as a prognostic value even in the context of tumor heterogeneity support its usefulness also in metastatic CRC.

## Methods

### Gene expression data

Gene expression profiles from 477 patients treated surgically for their primary CRC and/or liver metastases at Oslo University Hospital, Norway, between 2009 and 2019 were analyzed (Table 1). The original sample set consisted of 298 metastatic samples, 24 patient-matched non-malignant liver samples, and 317 primary tumor samples (from 315 patients^25,35,36^). Three metastasis samples with a low tumor cell content were discarded, based on an upper threshold for the “liver background” (described below) estimated in normal liver samples as reference (≤10th percentile). This retained 295 samples from 278 distinct liver metastatic lesions from 176 patients for further analysis. For intra-patient comparisons, matched primary-metastasis samples were available from 14 patients (76 samples and 59 lesions), and multiple metastatic deposits from each of 45 patients (169 samples), including from the 2nd and 3rd liver resections of 10 and 1 patients, respectively. All patients provided signed informed consent, and the study was conducted in accordance with the Declaration of Helsinki and approved by the Norwegian Data Protection Authority and Regional Committee for Medical and Health Research Ethics, South-Eastern Norway (REC numbers 1.2005.1629;2010/1805).

Previously published in-house gene expression data from 35 CRC cell lines and 24 patient-derived organoid cultures from resected liver metastases were used to identify genes with cancer cell-intrinsic expression^37,38^. Critically, all these different sample sets have been profiled for gene expression using the same technology (GeneChip Human Transcriptome Array 2.0; ThermoFisher Scientific, Waltham, MA, USA; according to the manufacturer’s instructions), to facilitate cross-sample type comparisons. Microarray data were processed using the R package *affy* (v1.66.0)^39^ with brainarray Entrez v24 CDFs^40^. ComBat method implemented in the R package *sva* (3.36.0) was used to account for batch effects from preprocessing.

A publically available gene expression data set including 545 primary CRCs and 73 metastatic lesions (GSE131418, “Consortium cohort”)^5^ was preprocessed with *affy* and used for validation analyses.

### MSI status and mutation analyses

Determination of MSI status (PCR‐based analyses of the BAT25/BAT26 markers or the five markers incorporated in the MSI Analysis System version 1.2 [Promega, Fitchburg, WI, USA]) and sequencing of *KRAS* and *NRAS* in exons 2–4, *BRAF* in exon 15 (including codon 600), and the full coding sequence of *TP53* (exons 2–11) has previously been performed on an Applied Biosystems 3730 DNA Analyzer (ThermoFisher Scientific)^41,42^.

### Development of CMS classifier adapted to CRC liver metastases

Three distinct steps were combined to develop a classifier tailored to liver metastases, including feature selection of the template gene set (similarly to our approach previously used to develop a classifier enriched with cancer cell-intrinsic features and suitable for pre-clinical models^13^), background adjustment to account for signals from the liver tumor microenvironment in bulk tissue samples, and training of a classifier based on the resulting template gene set in primary tumors with known CMS class. The workflow is outlined in Supplementary Fig. 5 and specified below.

#### Feature selection

Only protein-coding genes with unique 1:1:1 NCBI Entrez:GENCODE ensemble:HUGO Gene Nomenclature Committee mappings (*n* = 16,489) were considered, to facilitate portability. The following heuristics were used for feature selection in a combined gene expression matrix of CRC cell lines and patient-derived organoids (*n* = 59): (i) maximum expression value of the gene higher than the first tertile of the data set; and (ii) 10th–90th inter-percentile range of gene expression values among samples within the top 10%. These filters were used to enrich for robustly expressed and cancer cell-intrinsic genes, and retained 1104 genes as features for classification.

#### “Liver background” adjustment

To estimate the proportion of “liver background” in transcriptomic data from bulk tumor tissue samples, *P*, we leveraged the fact that hepatocytes have a high relative expression of specific gene markers (e.g., F9, *ALB*^16,43^). For sample *i* (*i* ∈ 1,2, ..., *N*), the proportion of “liver background,” *P*_*i*_, was estimated as shown in Eq. (1):

1 $$P_i = \frac{{p_i - p_{{\rm{min}}}}}{{p_{{\rm{max}}} - p_{{\rm{min}}}}}\,{\mathrm{where}}\,p_i = \mathop {\sum}\limits_{j = 1}^M {z_{ij}g_j}$$

Here, *g* represents all genes with gene $g_j(j \in 1,2, \ldots ,M)$ set to 1 if it is included in the Human Protein Atlas list of liver-specific markers, else 0. *z* represents the row/gene-wise mean-centered gene expression matrix. Ordinary least squares regression was used to solve *X* = *PB* and derive the expression of the cancer cell proper, *xc*, as the residuals *xc* = *X* − *PB*. Example R code is included in the following.

If *x* represents a gene expression matrix (gene in rows/sample in columns), the “liver background” was estimated and regressed out as follows:

*## R code*

*## liver background estimate*

x_centered <- **sweep**(x, 1, **rowMeans**(x))

g <- **ifelse**(**rownames**(x_centered) **%in%** liver_markers, 1, 0)

p <- **colSums**(x_centered[g,])

P <- (p - **min**(p)) / (**max**(p) - **min**(p))

*## regress out liver background*

fit <- **lm**(**t**(x)~P)

x_adj <- **t**(fit$residuals)

x_adj <- x_adj + **abs**(**min**(x_adj))

*P* is a vector of sample-wise estimates of the proportion of “liver background”, and x_adj is the background-adjusted gene expression matrix used for the classification of liver metastases.

#### Sample classification

CMS labels were assigned using the classifyCMS.RF function in the R package CMSclassifier (v1.0.0)^2^, using the default posterior probability of 0.5 to assign confident sample classifications. For primary CRCs, the resulting labels were considered “true” and used to train the tailored classifier.

Genes in the feature-selected template set were used as predictors to train a nearest shrunken centroids classifier on the gene expression data from CMS-labeled primary CRCs (*n* = 252 confidently classified tumors)^18^. The tuning parameter *t* was used to control the shrinkage of the data, and we used leave-one-out cross-validation to estimate the accuracy and determine the optimal tuning parameter. The resulting classifier was applied to the “background adjusted” liver metastases. The posterior probability threshold for confident sample classification was set to obtain a similar proportion of unassigned samples (NA) as initially reported^2^. This liver metastasis classifier was implemented as a PAM model. To provide a classifier that is not dependent on non-malignant liver samples, a random forest^44,45^ classifier was trained on the resulting labels and implemented in the lmCMScaller function. This model can be applied directly to liver metastases, with no need for “liver background” adjustment.

### Gene set analysis

Fourteen CRC- and CMS- informative gene sets (Fig. 2d) were retrieved as previously described^13^, and sample-wise gene set scores were calculated using the gene set variation analysis method implemented in the R package GSVA (v1.36.2)^15^.

### Statistical methods

All *p*-values were two-sided. Fisher’s Exact, Kruskal–Wallis, Pearson’s correlation, and Wilcoxon tests were performed using the functions fisher.test, kruskal.test, cor, and wilcoxon.test in the R package *stats*. PCA was performed using the R function prcomp on genes (*n* = 5000) with the largest cross-sample expression variance. Two-dimensional density estimates of principal component scores were computed using the function kde in R package *ks* (v1.11.7)^46^. General transcriptomic heterogeneity between pairs of samples from each patient was calculated as the pairwise Euclidean distance of the 3 first principal components (PC1–PC3 scores; dimensionality reduction was used to lower the challenge of high variance in gene expression data). R package *sinaplot* (v1.1.0) was used to add data points to boxplots^47^.

Kaplan–Meier and Cox proportional hazard analyses were performed for patients with liver metastases using the R package *survival* (v3.2–7). Hazard ratios, 95% CIs, and Wald tests were calculated using the coxph function. The primary end-point was median overall survival. Death from any cause was registered as an event and patients were censored at loss to follow-up or after five years. Time to event/censoring was calculated from the start of treatment, either date of surgical resection or neoadjuvant treatment.

### Reporting summary

Further information on research design is available in the Nature Research Reporting Summary linked to this article.

## Supplementary information

Supplementary Material

Reporting Summary

## Data Availability

Gene expression profiles of primary CRCs (*n* = 211; Gene Expression Omnibus accession numbers GSE79959, GSE96528, and GSE139170) and CRC cell lines (*n* = 34; GSE97023) have previously been published. Data from liver metastases (*n* = 283), including clinicopathological annotations, have been submitted under accession number GSE159216 (private until the publication of a separate manuscript). The remaining primary tumor samples have been submitted under accession number GSE178120. The validation data set was downloaded from GSE131418 (Consortium cohort).

## Code availability

The new CMS classifier tailored to liver metastases (random forest model) is implemented in the updated version of the R package CMScaller (v2.0.1) with the function lmCMScaller, and is available from https://github.com/Lothelab/CMScaller with documentation and user examples.
