# Supplementary material for: Metastatic heterogeneity of the consensus molecular subtypes of colorectal cancer
Source: NPJ Genom Med. 2021 Jul 14;6:59. doi: 10.1038/s41525-021-00223-7 (PMC8280229; doi:10.1038/s41525-021-00223-7)
Supplement: Supplementary file 1 — Supplementary Material [file 41525_2021_223_MOESM1_ESM.pdf]

Supplementary Figures 1 to 10 for:

Metastatic heterogeneity of the consensus molecular subtypes of colorectal cancer

Peter W. Eide, Seyed H. Moosavi, Ina A. Eilertsen, Tuva H. Brunsell, Jonas Langerud, Kaja C.G. Berg, Bård I. Røsok, Bjørn A. Bjørnbeth, Arild Nesbakken, Ragnhild A. Lothe, and Anita Sveen

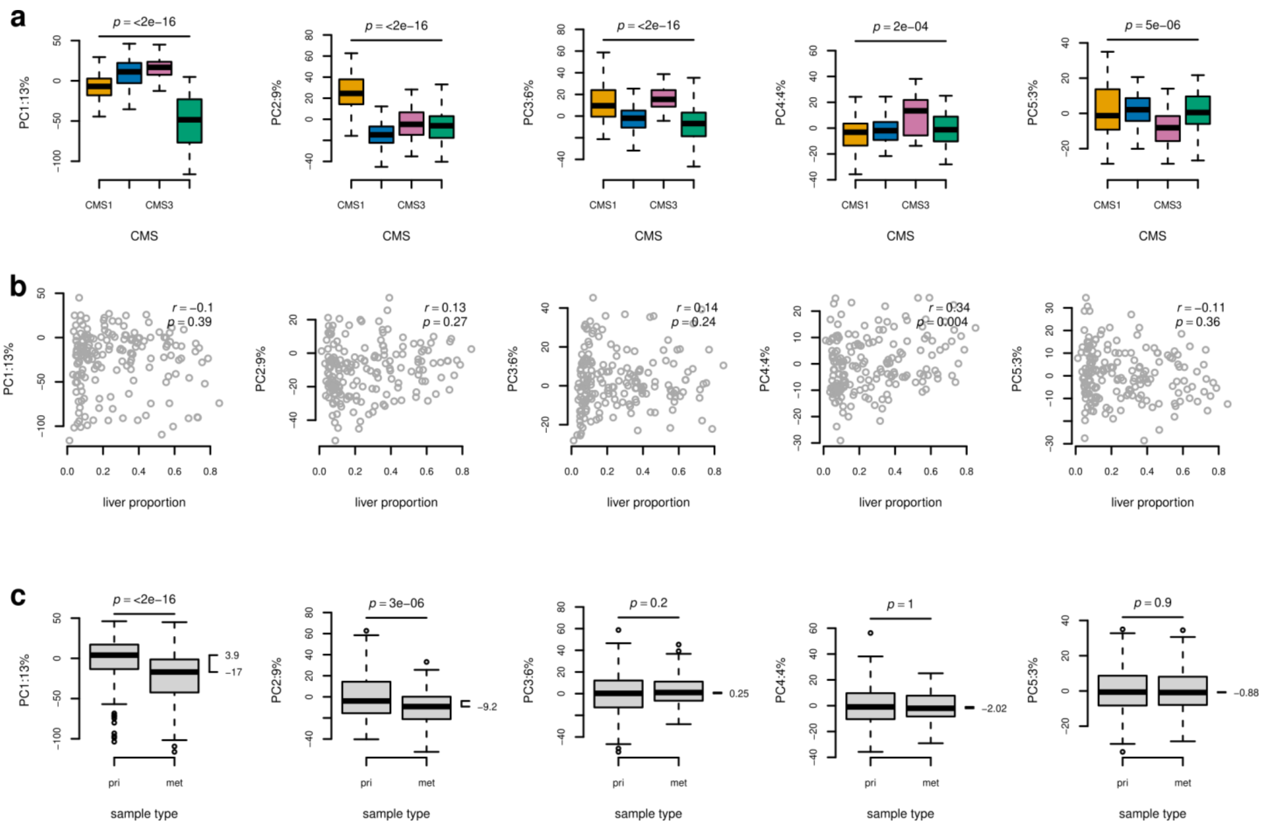

Supplementary Figure 1: PCA supporting that resected metastases represent a biased subset of primary CRCs related to CMS.

The first five principal components (PC1-PC5) among primary CRCs are (**a**) associated with the CMS of the primary CRCs (*p*-values from Kruskal-Wallis tests), (**b**) not associated with the estimated liver proportion in the primary tumor samples (Pearson correlation coefficients [*r*] and *p*-values are indicated), and (**c**) associated with sample type in comparisons of primary tumors and metastases (Wilcoxon test *p*-values and medians of PC scores are indicated). Boxplots: centre line, median; box limits, upper and lower quartiles; whiskers, 1.5× interquartile range.

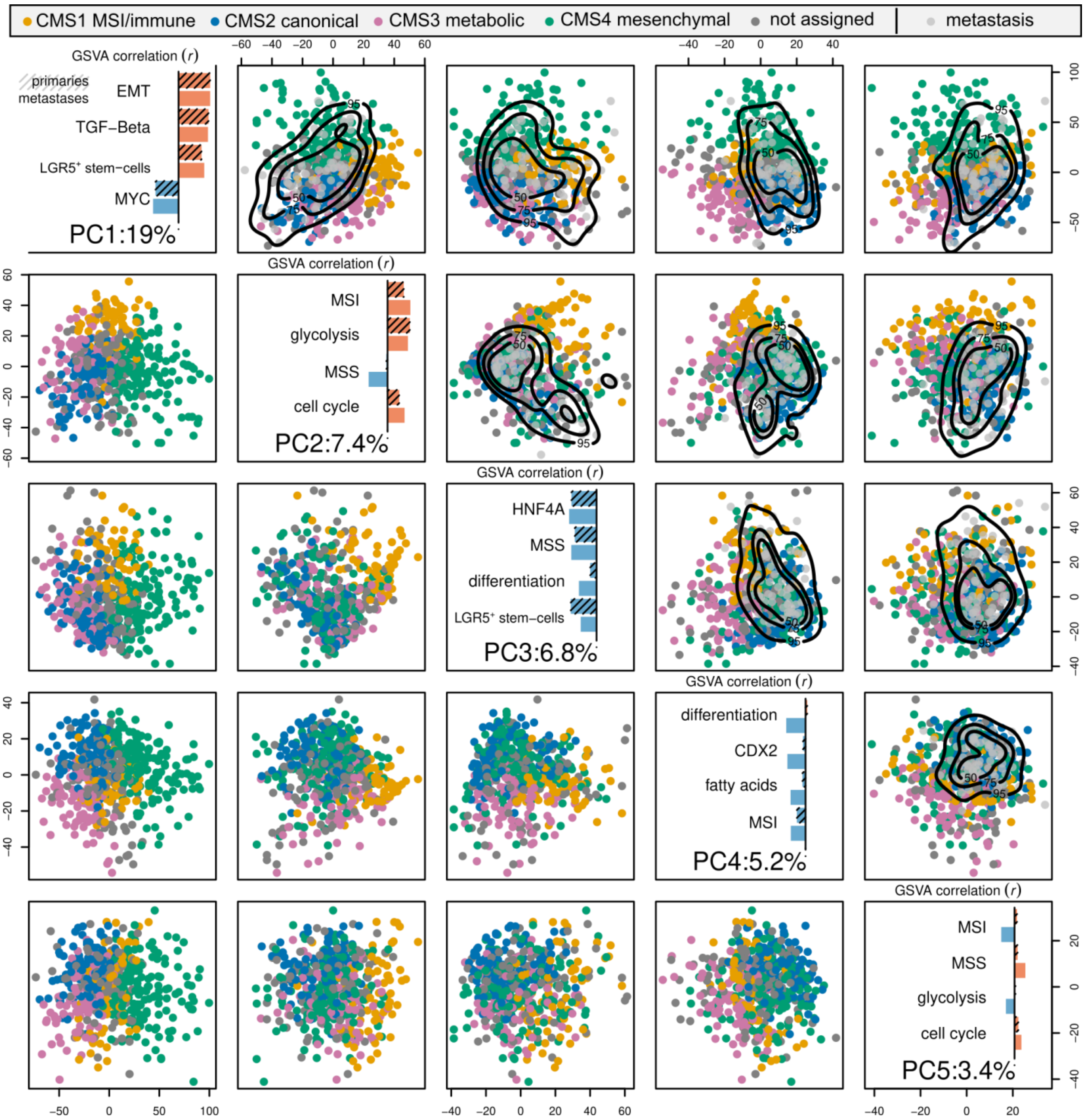

Supplementary Figure 2: Independent dataset [GSE131418] supporting that metastases have reduced variation along principal components separating CMS1/CMS3 primary tumors from CMS2/CMS4. The lower triangle of the scatterplot matrix depicts the first five principal components (PCs) for primary tumors, with samples colored according to CMS (dark gray=not assigned). The diagonal windows show the percentage of total variance explained by each PC (bottom) and the Pearson correlation (r) between the indicated PC and single-sample gene set scores (GSVA) for the top-4 correlated gene sets among primary tumors (blue and red represent negative and positive correlations, respectively). Correlations among liver metastases are shown for comparison. The upper triangle represents the transpose of the lower triangle with the metastatic samples (light gray) superimposed by projections onto the same PCs. Black contour lines represent the 50%, 75% and 95% 2D-density estimates.

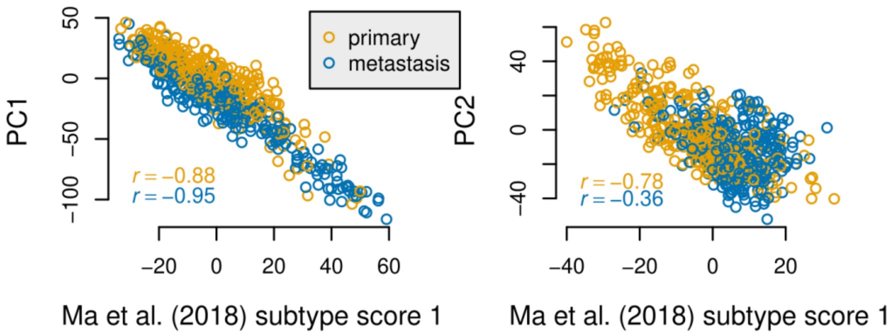

Supplementary Figure 3: High correlation between principal components and continuous subtype scores from Ma *et al*., Genome Biol. 2018; 19: 142.
Each point represents a sample colored according to sample type, and Pearson correlation coefficients (*r*) for primary tumors and metastases are indicated separately (*n*=612).

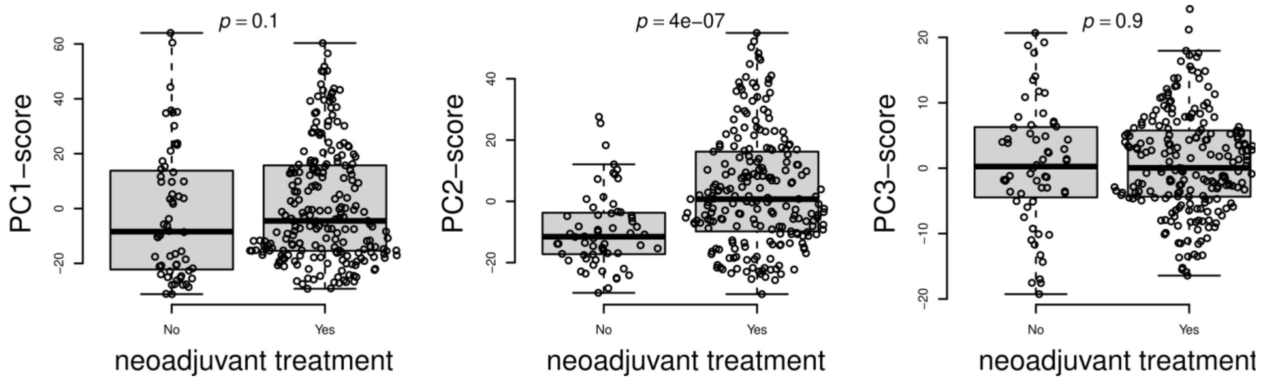

Supplementary Figure 4: Neoadjuvant chemotherapy is associated with principal components from gene expression profiles.

Principal components were derived from liver metastases only (*n*=295). Each point represents a sample and is grouped according to whether the patient had received neoadjuvant chemotherapy or not. *p*-values are from Wilcoxon-tests. Boxplots: centre line, median; box limits, upper and lower quartiles; whiskers, 1.5× interquartile range.

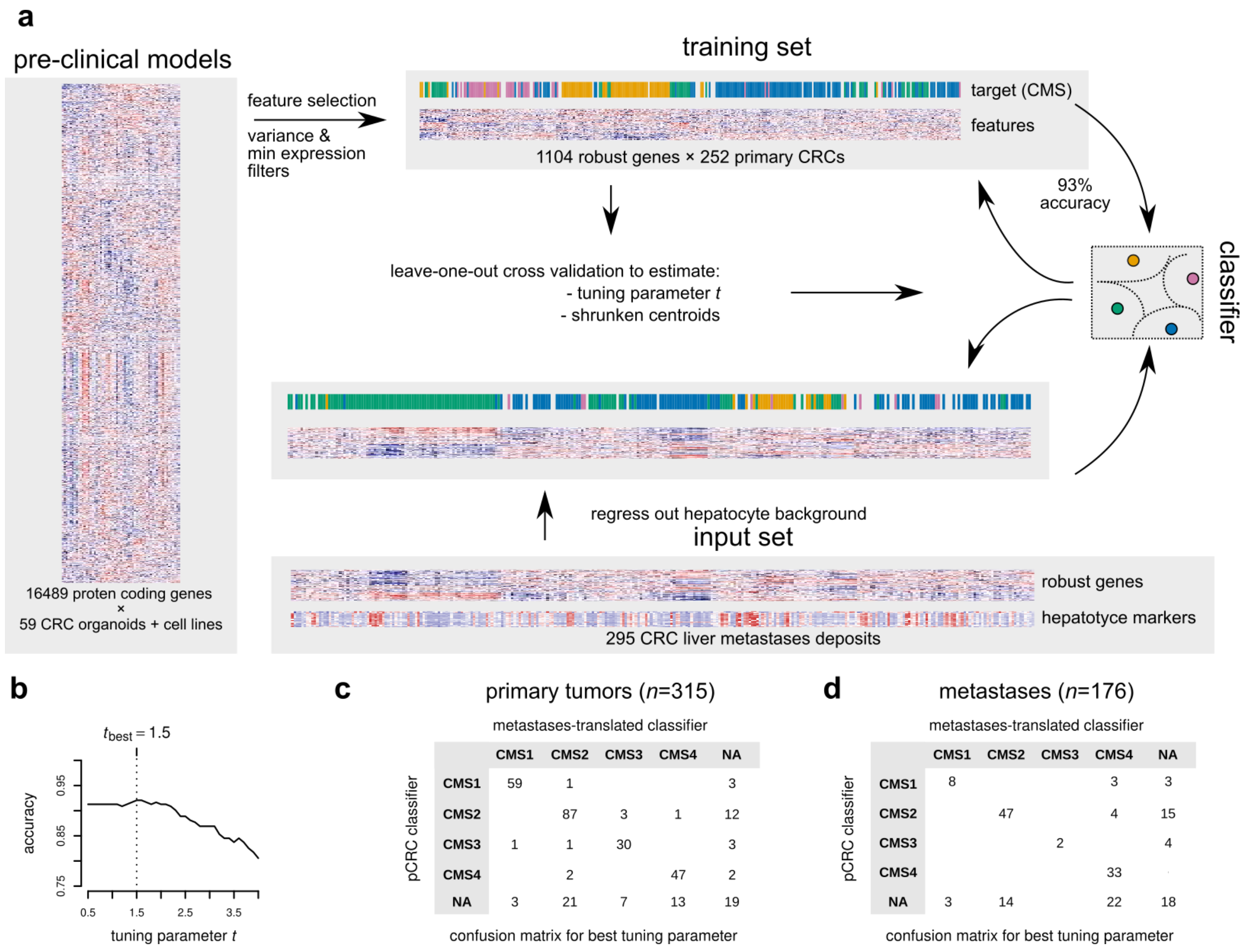

Supplementary Figure 5: Classifier parameter development, tuning and performance on training set.

(**a**) Overview of strategy for classifier development and data included. (**b**) Plot shows classification accuracy as a function of the tuning/regularization parameter *t* in primary tumors, with t_{best} indicated. (**c**) Confusion matrix indicates concordance in CMS classification of the primary CRCs (pCRC) using the original pCRC CMSclassifier and the resulting metastases-translated classifier (*n*=315 samples from unique patients). (**d**) Confusion matrix indicates concordance in CMS classification of the metastatic CRCs using the original pCRC CMSclassifier and the resulting metastases-translated classifier (*n*=176 samples from unique patients).

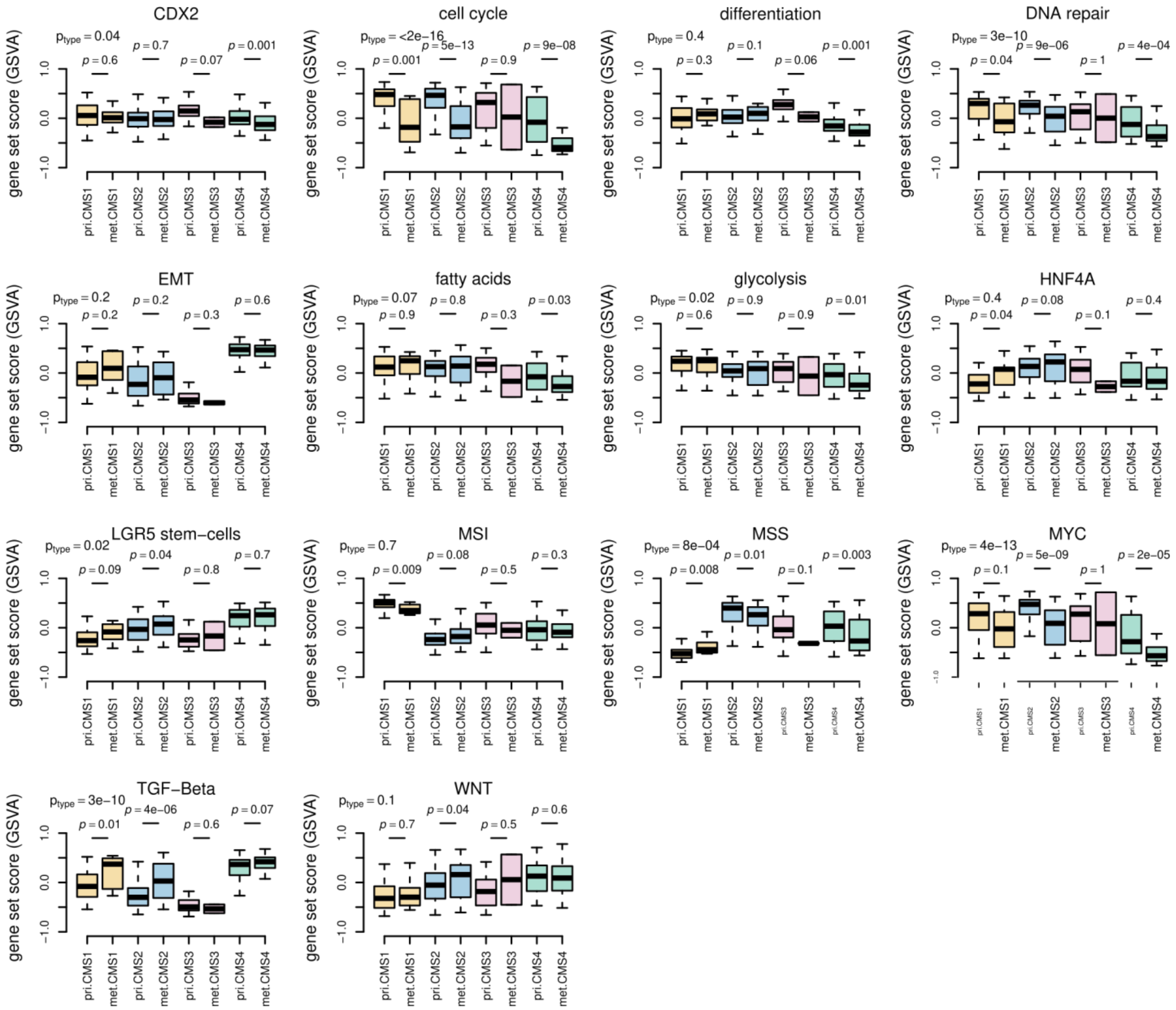

Supplementary Figure 6: Gene set associations for primary tumors and liver metastases. Boxplots (centre line, median; box limits, upper and lower quartiles; whiskers, 1.5× interquartile range) with single-sample GSVA scores for 14 pre-selected CRC- and CMS-relevant gene sets stratified by CMS and sample type (primary/metastasis). *p*-values are from Wilcoxon tests, comparing the two sample types within each CMS class. p_{type} refers to the *p*-value for sample type as a coefficient when GSVA scores are modeled as a function of CMS and tissue type.

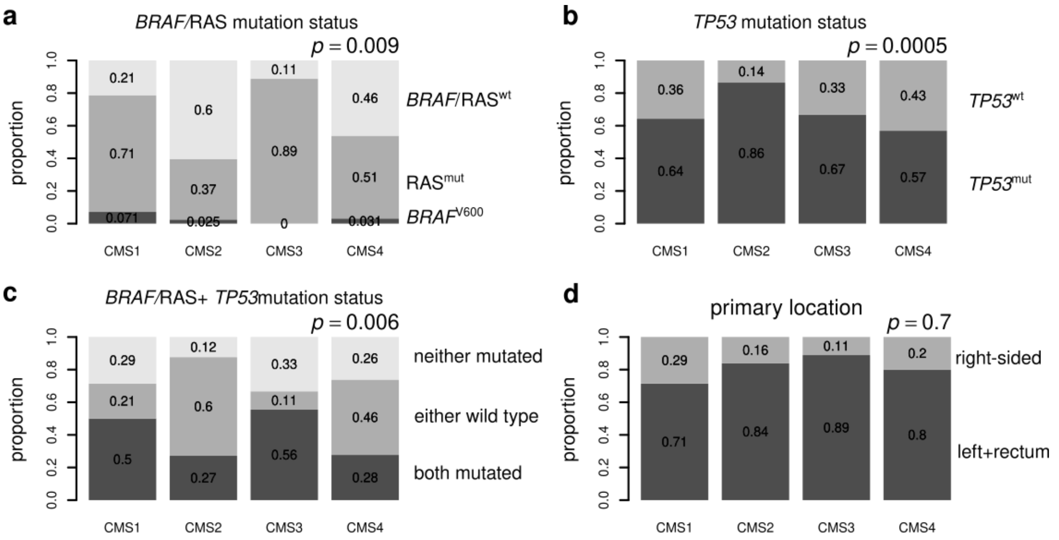

Supplementary Figure 7: Molecular and clinicopathological associations of CMS classified liver metastases.

Association between CMS and (**a**) *BRAF*^{V600} and *KRAS*/*NRAS* (RAS) hot-spot mutations, (**b**) *TP53* mutations, (**c**) co-occurrence of *TP53* mutations and *BRAF*/RAS hot-spot mutations and (**d**) location of primary tumor. *p*-values are from Fisher's tests. One unique sample per patient was included for these association analyses.

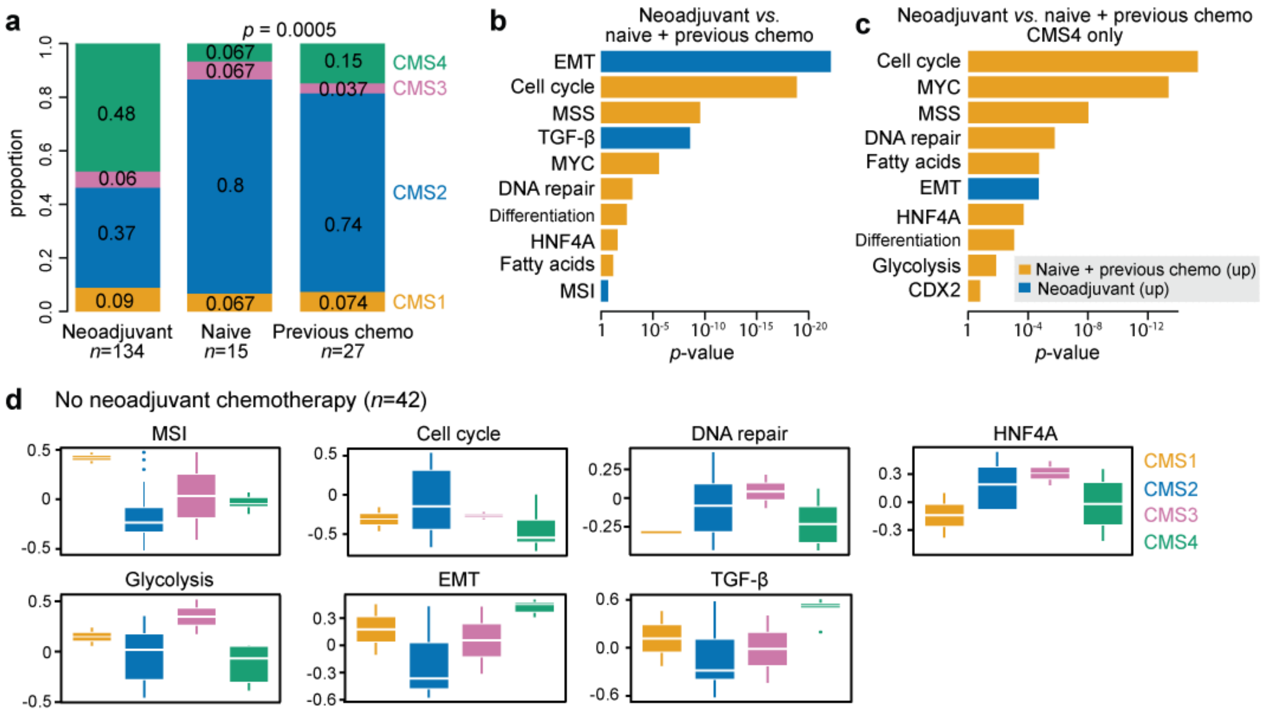

Supplementary Figure 8: Neoadjuvant chemotherapy is associated with CMS4 enrichment. (**a**) Barplots show association between chemotherapy exposure and CMS. Neoadjuvant represents patients who received neoadjuvant chemotherapy for the sampled metastases; naive represents patients who have not received any chemotherapy prior to tumor sampling; and previous chemo represents patients who received chemotherapy at any timepoint prior to sampling. *p*-value is from Fisher's test (*n*=176 patients). **(b-c)** Barplots show the top-10 among 14 pre-selected gene sets for camera gene set analysis comparing treatment groups as indicated (**b**) across all samples or (**c**) only samples classified as CMS4. (**d**) Boxplots (centre line, median; box limits, upper and lower quartiles; whiskers, 1.5× interquartile range) show single-sample enrichment scores (GSVA) of a selection of the gene sets according to the CMS of liver metastases from patients who did not receive neoadjuvant chemotherapy.

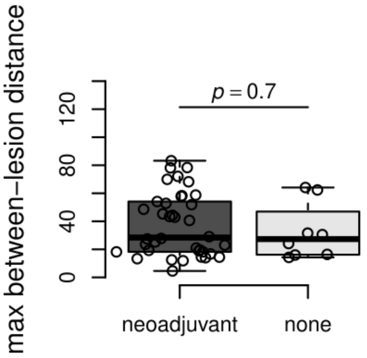

Supplementary Figure 9: No difference in maximum inter-lesion distances for patients according to neoadjuvant chemotherapy status.
Patients are compared with respect to the maximum inter-lesion distance, calculated as Euclidean distances in the three-dimensional PC1-PC3 space. *p*-value is from Wilcoxon test. Boxplots: centre line, median; box limits, upper and lower quartiles; whiskers, 1.5× interquartile range.

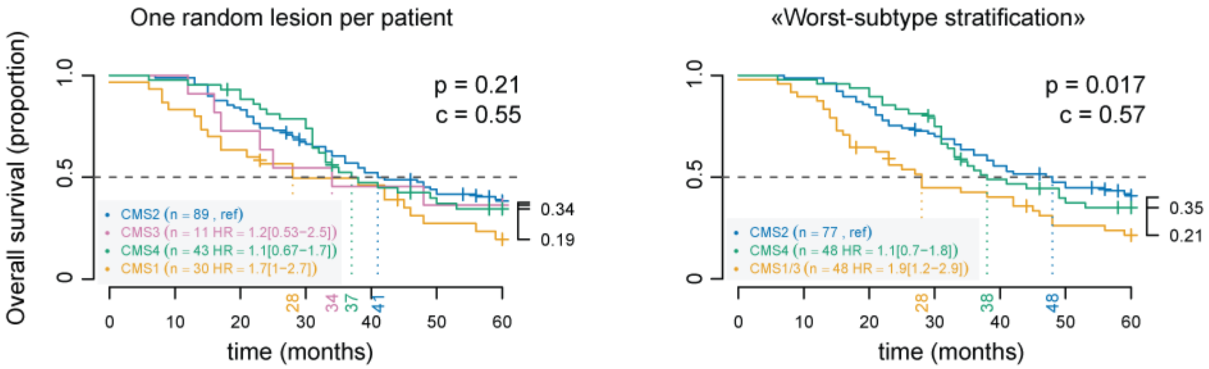

Supplementary Figure 10: Patient prognosis according to CMS classification with the original primary CMSclassifier.

Kaplan-Meier survival estimates for patients with resected liver metastases stratified by the CMS of one randomly selected lesion per patient (left) and if heterogeneous classifications, according to the “worst-subtype rule” (right): patients were assigned CMS1/CMS3 if any lesion was CMS1/CMS3; CMS4 if no lesion was CMS1/CMS3 and any was CMS4, and the remaining CMS2. *p*-values are from Wald-test, and *c* indicates concordance index. Samples were assigned to the nearest (not necessarily confident) CMS, and three patients with intermediate subtypes were excluded.
