# Supplementary material for: Metastatic heterogeneity of the consensus molecular subtypes of colorectal cancer
Source: NPJ Genom Med. 2021 Jul 14;6:59. doi: 10.1038/s41525-021-00223-7 (PMC8280229; doi:10.1038/s41525-021-00223-7)
Supplement: Supplementary file 2 — Reporting Summary [file 41525_2021_223_MOESM2_ESM.pdf]

nature research

Corresponding author(s): Anita Sveen
Last updated by author(s): May 18, 2021

Reporting Summary

Nature Research wishes to improve the reproducibility of the work that we publish. This form provides structure for consistency and transparency in reporting. For further information on Nature Research policies, see our Editorial Policies and the Editorial Policy Checklist.

Statistics

For all statistical analyses, confirm that the following items are present in the figure legend, table legend, main text, or Methods section.

n/a	Confirmed	
☐	☒	The exact sample size (n) for each experimental group/condition, given as a discrete number and unit of measurement
☐	☒	A statement on whether measurements were taken from distinct samples or whether the same sample was measured repeatedly
☐	☒	The statistical test(s) used AND whether they are one- or two-sided<br>*Only common tests should be described solely by name; describe more complex techniques in the Methods section.*
☐	☒	A description of all covariates tested
☒	☐	A description of any assumptions or corrections, such as tests of normality and adjustment for multiple comparisons
☐	☒	A full description of the statistical parameters including central tendency (e.g. means) or other basic estimates (e.g. regression coefficient) AND variation (e.g. standard deviation) or associated estimates of uncertainty (e.g. confidence intervals)
☐	☒	For null hypothesis testing, the test statistic (e.g. F, t, r) with confidence intervals, effect sizes, degrees of freedom and P value noted<br>*Give P values as exact values whenever suitable.*
☒	☐	For Bayesian analysis, information on the choice of priors and Markov chain Monte Carlo settings
☒	☐	For hierarchical and complex designs, identification of the appropriate level for tests and full reporting of outcomes
☐	☒	Estimates of effect sizes (e.g. Cohen's d, Pearson's r), indicating how they were calculated

Our web collection on statistics for biologists contains articles on many of the points above.

Software and code

Policy information about availability of computer code

Data collection	Microarray gene expression data were processed using the R package affy (v1.66.0) with brainarray Entrez v24 CDFs. ComBat method implemented in the R package sva (3.36.0) was used to account for batch effects from preprocessing, as described in the Methods section.
Data analysis	The manuscript describes the development of a computational method for classification of colorectal cancer liver metastases according to the consensus molecular subtypes. The method is implemented in an updated version of the R package CMScaller (v2.0.1), and will be available from GitHub (https://github.com/Lothelab/CMScaller) with documentation and user examples.

For manuscripts utilizing custom algorithms or software that are central to the research but not yet described in published literature, software must be made available to editors and reviewers. We strongly encourage code deposition in a community repository (e.g. GitHub). See the Nature Research guidelines for submitting code & software for further information.

Data

Policy information about availability of data

All manuscripts must include a data availability statement. This statement should provide the following information, where applicable:
- Accession codes, unique identifiers, or web links for publicly available datasets
- A list of figures that have associated raw data
- A description of any restrictions on data availability

Gene expression profiles of primary CRCs (n=211; Gene Expression Omnibus accession numbers GSE79959, GSE96528, and GSE139170) and CRC cell lines (n=34; GSE97023) have been published. Data from liver metastases (n=283), including clinicopathological annotations, have been submitted under accession number GSE159216 (private until publication of a separate manuscript). The remaining primary tumor samples will be deposited to the Gene Expression Omnibus and published in a separate manuscript. The validation dataset was downloaded from GSE131418 (“Consortium cohort”).

Field-specific reporting

Please select the one below that is the best fit for your research. If you are not sure, read the appropriate sections before making your selection.

☒ Life sciences ☐ Behavioural & social sciences ☐ Ecological, evolutionary & environmental sciences

For a reference copy of the document with all sections, see nature.com/documents/nr-reporting-summary-flat.pdf

Life sciences study design

All studies must disclose on these points even when the disclosure is negative.

Sample size

No sample size calculation was performed, and sample numbers were partly determined by availability. Totally 477 patients were included from the in-house series, and 317 primary tumor samples from 315 patients, as well as 298 samples from 278 liver metastatic lesions from 176 patients were analyzed. The datasets were representative of the general gene expression patterns of both primary and metastatic (resectable) colorectal cancers, and the primary tumors represented all four consensus molecular subtypes in an unbiased manner. Intra-patient inter-metastatic heterogeneity was analyzed in 169 samples of multiple metastatic lesions from each of 45 patients. Validation analyses were performed in an independent and publicly available set of 545 primary colorectal cancers and 73 metastases, confirming representativeness of the in-house patient series.

Data exclusions

Three metastasis samples with low tumor cell content were discarded from all analyses, based on an upper threshold for the "liver background" estimated in normal liver samples as reference (based on gene expression profiles, see Methods section for description). For selected analyses (indicated), only single patient-wise liver metastasis samples were included, for statistical independence. For intra-patient tumor heterogeneity analyses, only patients with multiple liver metastasis samples available were included.

Replication

Validation analyses were performed in a publicly available set of 545 primary colorectal cancers and 73 liver metastases, supporting that the metastases have a skewed transcriptomic distribution relative to primary tumors, as initially described in the in-house patient series.

Randomization

Randomization was not relevant to the study, the purpose was to classify/group samples/patients according to their intrinsic gene expression profiles.

Blinding

Blinding was not relevant to the study, but classification of samples according to their gene expression profiles was performed independently of clinicopathological or other molecular variables.

Reporting for specific materials, systems and methods

We require information from authors about some types of materials, experimental systems and methods used in many studies. Here, indicate whether each material, system or method listed is relevant to your study. If you are not sure if a list item applies to your research, read the appropriate section before selecting a response.

Materials & experimental systems

n/a	Involved in the study	
☒	☐	Antibodies
☐	☒	Eukaryotic cell lines
☒	☐	Palaeontology and archaeology
☒	☐	Animals and other organisms
☐	☒	Human research participants
☐	☒	Clinical data
☒	☐	Dual use research of concern

Methods

n/a	Involved in the study	
☒	☐	ChIP-seq
☒	☐	Flow cytometry
☒	☐	MRI-based neuroimaging

Eukaryotic cell lines

Policy information about cell lines

Cell line source(s)

This study involves re-analysis of a previously published gene expression dataset of CRC cell lines. A detailed description of the cell lines and their sources is included in the previous publication (Additional file 1: Table S1 in Berg et al., Mol Cancer 2017;16:116).
Cell line name Supplier Supplier ID
CaCo2 ATCC HTB-37
CL-11 DSMZ ACC 467
CL-34 DSMZ ACC 520
CL-40 DSMZ ACC 535
Co115 INSERM R. Hamelin, Paris
Colo205 ATCC CCL-222
Colo320 DSMZ ACC 144
Colo678 DSMZ ACC 194

DLD-1 ATCC CCL-221
EB INSERM R. Hamelin, Paris
FRI INSERM R. Hamelin, Paris
HCC2998 NCI60
HCT116 ATCC CCL-247
HCT15 ATCC CCL-225
HT29 ATCC HTB-38
IS1 INSERM R. Hamelin, Paris
IS3 INSERM R. Hamelin, Paris
KM12 NCI60
LoVo ATCC CCL-229
LS1034 ATCC CRL-2158
LS174T ATCC CL-188
NCI-H508 ATCC CCL-253
RKO ATCC CRL-2577
SW1116 ECACC 87071006
SW1463 ATCC CCL-234
SW403 ECACC 87071008
SW48 ATCC CCL-231
SW480 ECACC 87092801
SW620 ECACC 87051203
SW837 ATCC CCL-235
SW948 ECACC 91030714
TC71 INSERM R. Hamelin, Paris
V9P INSERM R. Hamelin, Paris
WiDr ATCC CCL-218

Authentication

Cell line authenticity has previously (Berg et al., Mol Cancer 2017;16:116) been verified by DNA profiling of 15 short tandem repeat loci, using the AmpFLSTR Identifiler PCR Amplification Kit (Thermo Fisher, Waltham, MA) and matched to the profiles from supplier where available.

Mycoplasma contamination

Cultures were tested for mycoplasma infection before collection using Myco Alert (Lonza, Walkersville, MD, USA) according to the manufacturer's protocol.

Commonly misidentified lines
(See ICLAC register)

WiDr is included along with the cell line it is found to be a derivative of, HT29. Gene expression data from the cell lines were analyzed collectively (also together with patient-derived organoids) across the complete set of samples, not sample-wise, and the impact of this particular cell line is therefore not likely to be strong.

Human research participants

Policy information about studies involving human research participants

Population characteristics

Clinicopathological characteristics of each of the patient series analyzed (primary and metastatic colorectal cancers) are provided in Table 1 in the manuscript.

Recruitment

Patients were recruited from observational studies of patients treated surgically for their primary colorectal cancer and/or colorectal liver metastases at Oslo University Hospital, Norway. Most primary rectal tumors treated with radiotherapy prior to sampling were excluded, to avoid the impact of treatment on gene expression profiles. Only samples with sufficient RNA quality were analyzed.

Ethics oversight

The study was approved by the Norwegian Data Protection Authority and Regional Committee for Medical and Health Research Ethics, South-Eastern Norway (REC numbers 1.2005.1629;2010/1805).

Note that full information on the approval of the study protocol must also be provided in the manuscript.

Clinical data

Policy information about clinical studies
All manuscripts should comply with the ICMJE guidelines for publication of clinical research and a completed CONSORT checklist must be included with all submissions.

Clinical trial registration

The study is not a clinical trial.

Study protocol

The study is not a clinical trial.

Data collection

Patients were recruited at Oslo University Hospital, Norway, between 2009 and 2019. Clinicopathological data and follow-up data were collected from hospital medical records.

Outcomes

Survival analyses for patients with colorectal liver metastases were performed with median overall survival as primary end-point. Death from any cause was registered as event and patients were censored at loss to follow-up or after five years. Time to event/censoring was calculated from start of treatment, either date of surgical resection or neoadjuvant treatment. This is described in the Methods section.
